# Translation, cross-cultural adaptation and psychometric evaluation of the Serbian Ankylosing Spondylitis Quality of Life (ASQoL) Questionnaire (refers to r-axSpA) and its relations with disease activity and functional status indices

**DOI:** 10.1186/s41687-025-00838-9

**Published:** 2025-01-15

**Authors:** Mirjana Zlatkovic-Svenda, Ana Djokic, Andjela Perunicic, Marija Zdravkovic, Slavica Pavlov Dolijanovic, Jeanette Thorpe, Dejan Dudok, Jelena Milicevic, Dejana Petrovic, Goran Radunovic

**Affiliations:** 1 Institute of Rheumatology, Belgrade, Serbia; 2 Faculty of Medicine, University of Belgrade, Belgrade, Serbia; 3 University of East Sarajevo Faculty of Medicine Foča, Republika Srpska, Bosnia and Herzegovina; 4 Clinical Hospital Bežanijska kosa, Belgrade, Serbia; 5 Galen Research Ltd, Manchester, UK; 6 General Hospital Pozega, Health Center Uzice, Uzice, Serbia; 7 Health Center Rakovica, Belgrade, Serbia

**Keywords:** Ankylosing spondylitis, Radiographic axial spondyloarthritis, Quality of life, ASQoL, Cross-cultural adaptation, Validation, Psychometric evaluation

## Abstract

**Objectives:**

To translate, cross-culturally adapt and validate the Serbian Ankylosing Spondylitis Quality of Life (ASQoL) questionnaire, e.g. according to the new nomenclature Radiographic-Axial Spondyloarthritis (r-axSpA), and to relate it to disease activity and functional status domains.

**Methods:**

Four stages were accomplished: (1) Bilingual and lay panel for translation and cross-cultural adaptation (2) Cognitive debriefing interviews (assessing the language and cultural equivalence of the concepts used in the Serbian ASQoL translation) for face and content validity (3) Psychometric evaluation: (a) convergent validity by NHP as a comparator scale and (b) known group validity by correlations with disease activity and overall health status and reliability (internal consistency and test-retest reliability) (4) Independent regression analyses for relations between ASQoL and ASDAS, BASDAI, BASFI, Schober’s test, respiratory index and SPARCC were used. The statistical program SPSS (version 21; IBM, Armonk, NY, USA) was used.

**Results:**

The bilingual panel made a unified version of the translated documents, a lay panel confirmed the clarity of the questionnaire. Cognitive debriefing interviews with 10 patients evaluated the Serbian ASQoL as clear, precise, easy to complete. The psychometric properties with 60 randomly selected patients showed good convergent validity between ASQoL and NHP domains of pain (*r* = 0.79), emotional reactions (*r* = 0.78), physical activity (*r* = 0.77) and energy (*r* = 0.75). The internal reliability was 0.95 and 0.91 (1st and 2nd administration), respectively, and the test-retest reliability was 0.84. Regression analyses showed highly significant relationships (*p* < 0.001) between ASQoL and ASDAS (R²=0.403), BASDAI (R²=0.564) and BASFI (R²=0.444).

**Conclusion:**

The Serbian ASQoL demonstrated good psychometric properties and significant relationships with disease activity and functional status and is recommended for quality of life assessment in Serbian-speaking ankylosing spondylitis (radiographic axial spondyloarthritis) patients, both in clinical practice and clinical research.

## Introduction

The Assessment of SpondyloArthritis International Society (ASAS) group has reached a new consensus on the nomenclature of axial spondyloarthritis (axSpA), with this term approved for both *radiographic* and *non-radiographic axSpA*, as the two forms refer to the same condition [1]. Additionally, the terms ankylosing spondylitis (AS) and radiographic axial spondyloarthritis (r-axSpA) should be used interchangeably, with preference given to the term r-axSpA in the future [1], which was implemented in this work for the first time for the QoL assessment.

The Ankylosing Spondylitis Quality of Life questionnaire (ASQoL) which is used for AS, according to the new nomenclature refers to radiographic-Axial Spondyloarthritis (r-axSpA).

R-axSpA is a chronic inflammatory rheumatic disease. It is more common in men than in women and usually begins before the age of thirty. Inflammation starts at the entheseal sites- the attachments of tendons, ligaments or capsules to the bones. Disease typically affects sacroiliac joints but can also spread upward to the lumbar and thoracic spine. The peripheral joints can be affected, and other organs, such as the heart, lungs and eyes, may occasionally be involved.

R-axSpA affects up to 1.4% of people. The estimated prevalence of spondyloarthritis in Serbia is 0.32%, with r-axSpA accounting for 0.08% of cases [2, 3]. The incidence and prevalence of r-axSpA are usually correlated with the presence of the HLA-B27 antigen [4].

If left untreated, r-axSpA may result in functional impairments that influence mood, everyday activities and motivation, causing social and occupational problems [5]. R-axSpA treatment objectives include low disease activity or remission, better quality of life and good functional status [6]. Quality of life (QoL) questionnaires are general and not designed for a specific rheumatic condition; for example, the EuroQoL 5D (*EQ-5D*) [7] and Short-form survey-36 (*SF-36*) [8] are specific to a particular disease.

Clinical trials frequently use the ASQoL for treatment efficacy assessments from the patient’s perspective [9, 10]. The questionnaire covers sleep, mood, motivation, independence, everyday activity, relationships and social life in r-axSpA patients. It was developed in the UK and the Netherlands and was adapted in several languages, demonstrating good psychometric properties [11–15].

The aim of this study was to translate, cross-culturally adapt and validate a Serbian version of the ASQoL, to relate it with disease activity and functional status indices and to approve its use in routine clinical practice and international clinical trials.

## Materials and methods

The process of ASQoL adaptation and validation involved three steps: (1) translation with cross-cultural adaptation, (2) cognitive debriefing interviews and (3) psychometric evaluation, using the Nottingham Health Profile (NHP) as a comparator scale. Additionally, the relationships of the ASQoL with disease activity and functional status indices were evaluated via independent regression analyses.

The ASQoL questionnaire contains 18 binary questions labelled “yes” or “no”, with scores ranging from 0 to 18, where a higher number indicates worse quality of life. The NHP comprises 38 items within six dimensions (sleep, physical mobility, pain, energy level, social isolation, and emotional reactions); these items are labelled binary with ‘yes’ or ‘no’, and each domain is scored from 0 (no issues or limitations) to 100 (every issue from the list is present) [15, 16].

### Translation with cross-cultural adaptation

The ASQoL questionnaire, along with all the instructions and supplemental materials, was translated from English into Serbian using the dual-panel methodology; a bilingual panel and a lay panel.

The bilingual panel included individuals with strong command of both Serbian and English and one person who participated in the original questionnaire development. Each group member translated each item into Serbian, and the unified version was agreed upon at a meeting. The lay panel consisted of monolingual Serbian speakers with low to average educational levels, who had to evaluate the draft version of the translation to assure that the expressions are in accordance with simple, everyday local language. They also agreed the final translations were easily understood by ordinary people. A range of ages should be represented in this group to ensure that the final measure is acceptable to all age groups.

### Cognitive debriefing interview

Cognitive debriefing interviews (CDIs) were used to evaluate the face and content validity of the Serbian ASQoL translation. ISPOR good practice guidelines state that 5–8 CDIs should be conducted, this study conducted 10 so that they represent a range of disease severity, gender and age as far as possible. The goal of the CDI is to assess the language and cultural equivalence of the used concepts, e.g. to estimate level of comprehensibility and cognitive equivalence of the translation, to test any translation alternatives that have not been resolved by the translators, to indicate any items that may be improper at the conceptual level and to identify any other issues that may cause misunderstanding [17].

Patients with r-axSpA completed the ASQoL questionnaire in the presence of a qualified rheumatologist who took notes about the acceptability, relevance and comprehensiveness of the questionnaire and recorded whether the patients had carefully read the instructions before filling it and whether they needed to go over the fundamental explanations again. Any potentially missed, distorted, ambiguous, unclear or difficult item was registered. A demographic questionnaire was given in addition to the ASQoL for basic demographic information.

### Psychometric evaluation

Regarding size of the sample used for validation, the Classical Test Theory can produce results with as little as 20 participants, but 50 or more is recommended for full psychometric testing [18].

Patients with r-axSpA were selected at random during an ordinary check-up at the Institute of Rheumatology in Belgrade between June 1 and December 31, 2016.

The Serbian ASQoL questionnaire was completed twice, at two-week interval, under the same circumstances and at the same location. During the first visit, participants also completed the Nottingham Health Profile (NHP) and the demographic questionnaire.

Construct validity was tested by convergent validity, which shows associations between the ASQoL and the NHP questionnaire. Known group validity evaluates the capacity of the ASQoL to distinguish between patients with varying perceptions of overall health status and disease severity.

Internal consistency was assessed using Cronbach’s alpha coefficient, which measures the interrelations between items to form a scale and is accepted if it is above 0.7 [19]. Test-retest reliability measures reproducibility over time and helps in evaluating degree to which external factors affect the test scores [20].

### Relations with disease activity and functional status indices

During the first visit, disease activity and functional capacity were measured using the Ankylosing Spondylitis Disease Activity Score (*ASDAS*), Bath Ankylosing Spondylitis Disease Activity Index (*BASDAI*), Bath Ankylosing Spondylitis Functional Index (*BASFI*), Schober’s test, respiratory index, and Spondyloarthritis Research Consortium of Canada Enthesitis (*SPARCC*) index.

### Statistical analysis

In addition to descriptive statistical methods, Spearman’s rank correlation coefficient was used for convergent validity, the Mann‒Whitney U test was used for known-group validity for two groups, and the Kruskal‒Wallis test for three or more groups. For relations between ASQoL and ASDAS, BASDAI, BASFI, Schober’s test, respiratory index and SPARCC independent regression analyses were used. Based on previous experiences, the sample size was proven to be sufficient for psychometric evaluation. The statistical program SPSS (version 21; IBM, Armonk, NY, USA) was used; a *p* value at least 0.05 was considered significant.

This study was approved by the Ethical Committee of the Rheumatology Institute (No 29/1–62).

## Results

### Translation and cross-cultural adaptation

The dual panel translation methodology, which is recommended for needs-based instruments, was employed in this study. It involves the use of two independent translation panels: a Bilingual panel and a Lay panel.

The bililingual panel comprised 6 people (three males) aged 27 to 58 years, mean (SD) age 38.3 (10.9) years (one company director, one student, one food technician, one librarian and two unemployed persons), who were fluent in both English and Serbian, with Serbian being their mother language. Preference in recruitment was given to individuals who do not have a clinical background. The panel translated the ASQoL questionnaire and the accompanying documents as a group, this included the front page instructions, the instructions throughout, the items and response options into Serbian. The members discussed each translation in order to make consensus on the final translation, where an approximate English equivalent of the Serbian text (e.g. back translation) was also produced in order to check the original meaning and to preserve the conceptual essence. This meeting was also attended by a representative from Galen Research (JTh), the original development team of the ASQoL, who is a native UK English speaker. They advised on all item’s conceptual meanings.

Two problematic items discussed in detail **were**: Item 6 which was translated into two ways *“I rarely see my friends and family”* and *“I can’t participate in hanging out with my friends and family”*, with the latter one being preferred by the bilingual panel, because the term ‘hanging out’ represents deeper relationships for Serbians. Also, Item 10: *“It takes a long time to get going in the morning”* was translated into *“It takes me a long time to get moving in the morning”*, since the moving stands for ‘get going’ in Serbian. There were also two items (items 7 and 15) that required more discussion in the lay panel.

The lay panel included six adults (three males) aged 25 to 60 years, with a mean (SD) age 37.7 (14.9) years and an average to low educational level (a cashier, labourer, police officer, retiree, and two unemployed persons). Individuals with higher (university/ college) education or higher professional qualifications were excluded from the panel. Patients were also excluded from this panel, as its purpose was to determine the most appropriate wording for the questionnaire, rather than to comment on the appropriateness of the items. All participants were mono-lingual and spoke only Serbian. A range of ages was represented in this group, with at least one younger and one older (post-retirement) participant to ensure that the final measure is acceptable to all age groups. Without reviewing the original English ASQoL questionnaire, the lay panel evaluated the bilingual panel’s translations and reached an agreement on all issues at a focus-group discussion.

Items 7 and 10 were confirmed by the lay panel to be correct and no changes were made. Item 15 was checked with the lay panel, who suggested changing it to *‘I feel like I’m missing out on a lot in life’* to match the original English item better, this change was accepted. Items 4, 14 and 18 were amended slightly to be closer to everyday Serbian language. For item 6 the lay panel chose the same translation that was preferred by the bilingual panel, translated as ‘*I can`t participate in hanging out with my friends and family’*. Item 11 was amended sightly to portray a ‘feeling’, translated as *‘I don’t feel capable of doing housework’*.

### Cognitive debriefing interview

Cognitive Debriefing Interviews (CDIs) were conducted with 10 radiographic axial spondyloarthritis patients (five men) with a mean age (SD) of 38.4 (8.8) years (range 23 to 45) at the Institute of Rheumatology in Belgrade from January 20th to February 4th 2016 (Table 1). All CDIs were completed individually and face-to-face, by the same interviewer who followed the standardized interview methodology. Interviews were conducted in Serbian, the native language of the interviewer and the mother tongue of all patients.

**Table 1:** Cognitive debriefing interviews and psychometric evaluation: demographic information of respondents

	Cognitive debriefing interviews (*N* = 10)	Validation survey (*N* = 60)
Gender N (%)		
Male	5 (50.0)	42 (70.0)
Age (years) mean (SD)	38.4 (8.8)	38.2 (11.2)
Range	23–45	19–61
AS duration (years) mean (SD)	/	7 (6.3)
Residential status, N (%)		
Married/Cohabiting	5 (50.0)	31 (51.7)
Divorced/Widowed/Single	5 (50.0)	29 (48.3)
Working status N (%)		
Working full-time	/	31 (51.7)
Retired	/	6 (10.0)
Student	/	6 (10.0)
Homemaker	/	4 (6.7)
Long-term sick leave	/	2 (3.3)
Unemployed	/	11 (18.3)
Disease activity, mean (SD)		
BASDAI	/	2.5 (1.9)
ASDAS	/	1.9 (1.2)
Functional indices, mean (SD)		
BASFI	/	2.5 (2.1)
SPARCC	/	0.5 (1.2)
Schober’s test	/	4.2 (1.5)
Respiratory index	/	3.9 (1.8)
Perceived AS severity N (%)		
Mild	3 (30.0)	32 (53.3)
Moderate	4 (40.0)	16 (26.7)
Severe	3 (30.0)	12 (20.0)
Perceived general health N (%)		
Very good	1 (10.0)	14 (23.3)
Good	4 (40.0)	30 (50.0)
Fair	5 (50.0)	13 (21.7)
Poor	0 (0)	3 (5.0)

*ASQoL* Ankylosing Spondylitis Quality of Life Questionnaire, *BASDAI Bath Ankylosing Spondylitis Disease Activity Index*, *ASDAS Ankylosing Spondylitis Disease Activity Score*,* BASFI Bath Ankylosing Spondylitis Functional Index*,* SPARCC* SpondyloArthritis Research Consortium of Canada

All respondents understood the questionnaire directions and the purpose of the interview. The average time to complete the questionnaire was 3.1 min (range 2 to 6 min). There were no omitted or skipped items, confusing or unsatisfactory questions, or items that the patients were unable to answer. The Serbian version of the ASQoL questionnaire was judged as concise, easy to understand and complete. All of the questions were found appropriate. All participants carefully read the instructions before completing, and no one returned to them during the filling. All the individuals agreed that the questionnaire accurately assessed their condition. However, a few items were regarded as perplexing or ambiguous. The original measure developer was consulted on the ambiguous questions.

Some items were discussed in more detail: Item 7, translated as *“I am chronically tired”*, three respondents believed it was vague and used a more technical word, so the term *“chronically”* was replaced with *“always”*. Item 9, that was translated as *“I have unbearable pain”*, two respondents found that it is not precise enough, so the term *“unbearable”* was replaced with the more appropriate expression *“unendurable”*. The meanings of both expressions in the Serbian language are the same, but the new expression is used mainly to describe pain and feels more natural in Serbian. Item 11, translated as *“I don’t feel capable of doing housework “*, two patients were perplexed by the negotiation setting which is not common in Serbian, so this was changed to *“I feel incapable of doing the housework”*. Item 13, translated as *“I often get frustrated”*, three patients did not understand the meaning of the Serbian word for *“frustrated”* because this term is not commonly used in everyday language, but mostly in literature; therefore, it was changed to *“dissatisfied”*. Item 14, translated as *“The pain is always there”*, four patients felt that the phrase *“always”* was insufficient because they experienced pain *“often”* or *“occasionally”*, however, the item was not changed because removing ‘always’ would change the severity of the item. The instructions on the first page were also amended slightly to make them clearer.

### Psychometric evaluation

The Serbian ASQoL was validated in 60 patients (42 men; 70%) treated at the Institute of Rheumatology in Belgrade. The mean age of responders was 38.2 years (SD 11.2), range from 20 to 61 years. Around half of the sample (51.7%) were married or cohabiting, 51.7% were employed, self-perceived disease severity was rated as mild by 53.3% of respondents, and self-perceived general health status was rated as good by 50% (Table 1).

**Fig. 1:**
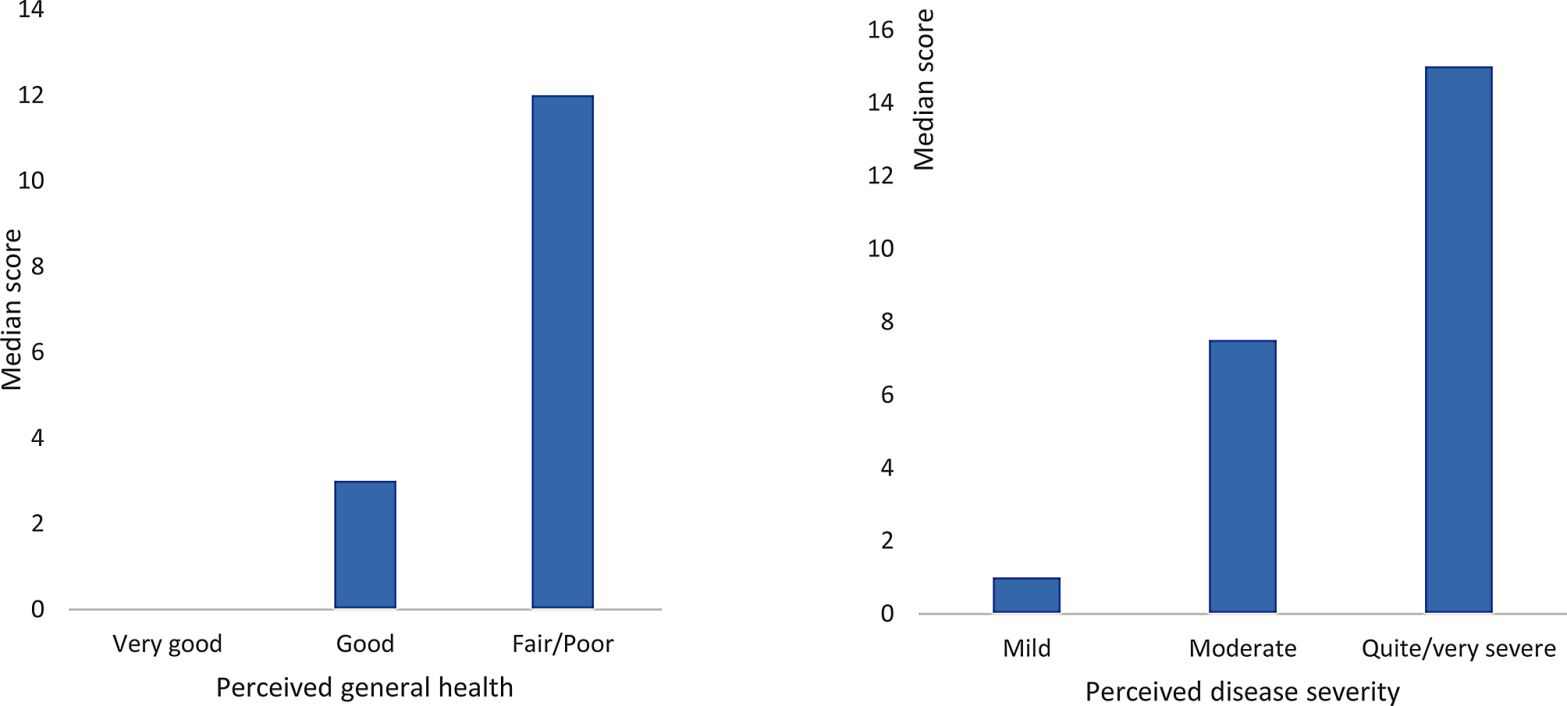
Median ASQoL scores according to patient-perceived general health and disease severity. *Note* All correlations are significant at the *p* < 0.001 level (2-tailed)

The ASQoL strongly correlated with pain (*r* = 0.79), emotional reactions (*r* = 0.78), physical activity (*r* = 0.77) and energy (*r* = 0.75) scales of the NHP. Cronbach’s alpha was 0.95 at administration 1 and 0.91 at administration 2, indicating high internal reliability (Table 2). With a Spearman correlation coefficient of 0.84, the test-retest reliability was strong, and the random error was low. The Mann‒Whitney U test showed statistically significant differences in ASQoL scores according to patient general health and disease severity (both *p* < 0.001) (Fig. 1).

**Table 2:** Correlations between the Serbian version of the ASQoL and different domains of the NHP

	Range	Median	IQR	% of patients scoring min	% of patients scoring max	Correlation coefficients with ASQoL Administration 1	Cronbach alpha coefficient
ASQoL Administration 1 (*n* = 60 patients)	0–18	3.5	1.0–10.0	23.3	1.7	-	**0.95**
NHP							
Energy level	0–100	33.3	0–66.7	48.3	20.0	0.75	-
Pain	0–100	18.8	0–62.5	38.3	8.3	0.79	-
Emotional reactions	0–88.9	0	0–22.2	53.3	0	0.78	-
Sleep	0–100	0	0–40.0	55.0	5.0	0.52	-
Social isolation	0–80	0	0–0	80.0	0	0.50	-
Physical mobility	0–62.5	12.5	0–37.5	33.3	0	0.77	
ASQoL Administration 2 (*n* = 59 patients)*	0–18	3.0	0–8.0	25.4	1.7	**0.84**	**0.91**

*Note* All correlations are significant at the 0.01 level (2-tailed)

*ASQoL* Ankylosing Spondylitis Quality of Life Questionnaire, *NHP* Nottingham Health Profile

*ASQoL Administration 2, patients who came for the second ASQoL filling, two weeks later

Bolded: correlation coefficient between ASQoL time 1 and ASQoL time 2; Cronbach alpha coefficient for ASQoL administration 1 and ASQoL administration 2

### ASQoL, disease severity and functional status indices

**Fig. 2:**
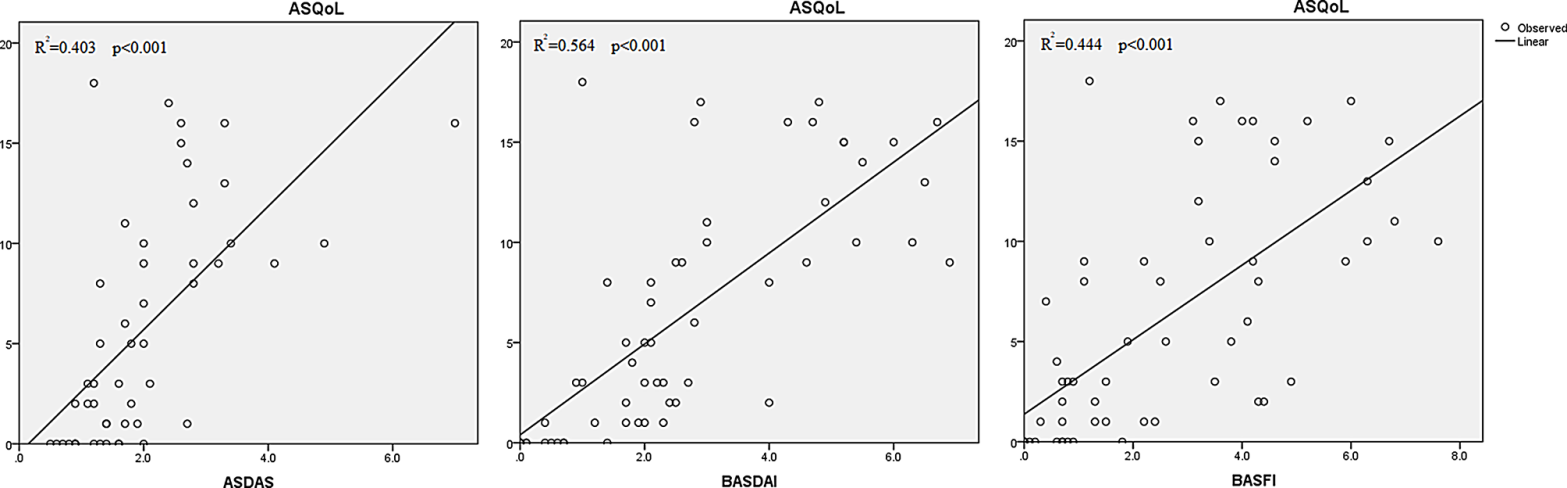
Regression analyses showing the relationships between the ASQoL and ASDAS, BASDAI and BASFI. *ASQoL* Axial Spondyloarthritis Quality of life, *ASDAS* Ankylosing Spondylitis Disease Activity Score, *BASDAI* Bath Ankylosing Spondylitis Disease Activity Index, *BASFI* Bath Ankylosing Spondylitis Functional Index

Independent regression analyses that set ASDAS, BASFI, BASDAI, SPARCC, respiratory index, Schober’s test as predictors and ASQoL as an outcome variable revealed positive relations of ASQoL with ASDAS- moderate (R^2^ = 0.403, F = 32.3; DF1 = 1, DF2 = 48; unbeta 3.078, *p* < 0.001); with BASDAI- strong (R^2^ = 0.564, F = 74.9, DF1 = 1, DF2 = 58; unbeta 2.268, *p* < 0.001) and with BASFI- moderate (R^2^ = 0.444, F = 46.4, DF1 = 1, DF2 = 58, unbeta 1.859, *p* < 0.001) (Fig. 2). Relations between ASQoL and SPARCC as well as between ASQoL and respiratory index were also significant but unacceptable, given the low coefficient of correlation (R^2^ = 0.091, F = 5.78; DF1 = 1, DF2 = 58; unbeta 1.488, *p* = 0.019; R^2^ = 0.114, F = 7.47; DF1 = 1, DF2 = 58; unbeta − 1.267, *p* = 0.008, respectively). There were no relations between the ASQoL and Schober’s test (R^2^ = 0.018, F = 1.04; DF1 = 1; DF2 = 58; non-beta-0.446, *p* = 0.312).

## Discussion

The ASQoL is a disease-specific measure which was created from qualitative interviews with patients. The translation with cross-cultural adaptation and psychometric evaluation of the Serbian ASQoL questionnaire was successfully completed.

The dual-panel methodology was used for translation, as it is recommended for patient-reported outcomes (PROs) [21, 22]. Following the CDIs some items (7, 9, 11 & 13) required modifications to improve or simplify the translation.

Out of three items that were passed from the bilingual panel on to the lay panel as they needed more discussion (Item 7, 10 and 15), one was afterwards changed by the CDIs: Item 7, where the term *“chronically”* was not easily understood and was therefore replaced by *“always”*. Items 9, 11 & 13 were also slightly changed to make them simpler in Serbian.

Generic PROs such as the SF-36 and EQ-5D are non-specific and are commonly used to assess QoL in patients with rheumatic diseases in general. However, these methods have significant limitations and drawbacks- weak psychometric qualities and failure to capture all essential characteristics of the particular disease and to determine substantial changes over time [23, 24]. The disease cannot be separated from personal and social aspects, so the health-related quality of life (HRQoL) questionnaires are useful measures for the overall treatment outcomes [25, 26].

According to the latest ASAS/EULAR (Assessment of SpondyloArthritis international Society/European League Against Rheumatism) recommendations for treating the patient with axSpA, the primary goal is to maximize long-term health-related quality of life through control of symptoms and inflammation, prevention of progressive structural damage and preservation/normalization of function and social participation [27].

The ASQoL is developed through qualitative interviews with patients, therefore all of its items are specifically relevant to AS (r-axSpA) patients. The metric was built on a well-defined theoretical construct, the needs-based model of QoL. These are both necessary prerequisites for QoL tools in clinical research [28].

The Serbian ASQoL questionnaire has shown good psychometric properties, with high levels of internal consistency, test-retest reliability and construct validity. The internal consistency tested with Cronbach’s α (0.95 and 0.91) was similar to that of the original ASQoL (0.91) and to that of other rheumatic diseases, such as the RAQoL (0.92) and PSS QoL (0.92) [15, 29, 30]. According to a systematic review article with meta-analysis, the test-retest reliability correlation coefficient of the ASQoL questionnaire in AS (r-axSpA) patients and nr-axSpA patients was 0.85 (95% CI 0.80 to 0.89) [31].

The ASQoL demonstrated good construct validity by correlating with pain, emotional reactions, physical activity and energy scales of the NHP questionnaire and good ability to detect substantial changes in different stages of the general health status and disease severity.

PROMs widely used in r-AxSpA such as the Bath Indices (BASDAI, BASFI and BAS-G) provide restricted information on the impact of r-axSpA and its treatment on the patient [12]. Although PROMs are not always substantially associated with disease severity in clinical practice, our study has found significant positive relations of ASQoL with disease activity and functional status in r-axSpA (AS) patients, e.g. moderate relations with ASDAS, strong relation with BASDAI and moderate relation with BASFI, which underlines the importance of patient-centred care, that provides a more personalized and successful approach to r-axSpA (AS) management [32].

This is the first study that has used regression analyses for relationships between ASQoL and disease activity and functional status in r-axSpA patients. According to the literature, correlations were used so far for this evaluation, but there were no studies examining correlations between ASQoL and ASDAS. Otherwise, mild-to-moderate correlation was observed between the Turkish version of ASQoL and BASDAI, BASFI and VAS-pain [14], a moderate-to strong correlation between the French version of ASQoL and the above-mentioned parameters [33] and between Chinese version of ASQoL and BASFI, VAS-pain and BASDAI total [34]. Moreover, a strong correlation was observed between ASQoL and BASDAI, BASFI and VAS-pain in New Zealand [35] as well as between ASQoL and BASDAI, BASFI in Turkey [36].

This study has a few limitations. The scaling features of the measurements could not be evaluated because Rasch analysis requires a larger sample size than was provided [37, 38]. Additionally, because this study did not include an intervention, the responsiveness of the Serbian ASQoL could not be assessed.

A further study is needed to determine whether the Serbian ASQoL can measure change in QoL in longitudinal studies and collect data that will allow scores (and changes in scores) to be interpreted in terms of clinical importance.

Internal measurement validity could also be assessed using a simple one parameter logistic IRT model—the Rasch model, with a larger sample size, recommended 250 or more. The Rasch model is a probabilistic model that states that an item response is a result of an interaction between person ability (e.g., level of mobility) and item difficulty. If data fit the model, the scale is defined as being unidimensional, meaning all items capture a single underlying domain (QoL) and can be combined to derive a valid total score.

There were no missing data in the current study. The ASQoL questionnaire was completed at the Institute of Rheumatology in the presence of young doctors, however individuals were requested to complete it independently with no input from the doctor. Clinical measurements were performed at the Clinical Measuring ambulance at the Institute of Rheumatology by the medical staff, taking all of the measurements. For Administration 1, 60 ASQoL were entirely completed, but Administration 2 only had 59, which is more than enough to undertake test re-test analysis.

## Conclusion

The Serbian ASQoL has demonstrated good psychometric properties and reliability for assessing quality of life in r-axSpA patients, and it can now be used in clinical practice, research, and national and international clinical studies for Serbian-speaking patients with r-axSpA (AS).

## Data Availability

The datasets used and/or analysed during the current study are available from the corresponding author on reasonable request.

## Abbreviations

ASQoL: Ankylosing Spondylitis Quality of Life
HRQoL: Health-related quality of life
EQ-5D: EuroQoL 5D
SF-36: Short-form survey
axSpA: Axial Spondyloarthritis
r-axSpA: Radiographic axial Spondyloarthritis
nr-axSpA: Non-radiographic axial Spondyloarthritis
ASAS: Assessment of SpondyloArthritis international Society
EULAR: European League Against Rheumatism
NHP: Nottingham Health Profile
CDI: Cognitive Debriefing Interview
PROs: Patient-reported outcomes
ASDAS: Ankylosing Spondylitis Disease Activity Score
BASDAI: Bath Ankylosing Spondylitis Disease Activity Index
BASFI: Bath Ankylosing Spondylitis Functional Index
SPARCC: Spondyloarthritis Research Consortium of Canada Enthesitis
SPSS: Statistical Package for Social Sciences
